# Recurrent aphthous stomatitis may be a precursor or risk factor for specific cancers: A case‐control frequency‐matched study

**DOI:** 10.1002/cam4.1685

**Published:** 2018-07-15

**Authors:** Lei Qin, Yi‐Wei Kao, Yueh‐Lung Lin, Bou‐Yue Peng, Win‐Ping Deng, Tsung‐Ming Chen, Kuan‐Chou Lin, Kevin Sheng‐Po Yuan, Alexander T. H. Wu, Ben‐Chang Shia, Szu‐Yuan Wu

**Affiliations:** ^1^ School of Statistics University of International Business and Economics Beijing China; ^2^ Graduate Institute of Business Administration Fu Jen Catholic University Taipei Taiwan; ^3^ School of Mathematical Sciences University of Nottingham Ningbo China Ningbo China; ^4^ Department of Dentistry Taipei Medical University Hospital Taipei Taiwan; ^5^ Graduate Institute of Biomedical Materials and Engineering Taipei Medical University Taipei Taiwan; ^6^ Department of Otorhinolaryngology Shuang‐Ho Hospital Taipei Medical University Taipei Taiwan; ^7^ Department of Oral and Maxillofacial Surgery Wan Fang Hospital Taipei Medical University Taipei Taiwan; ^8^ Department of Otorhinolaryngology Wan Fang Hospital Taipei Medical University Taipei Taiwan; ^9^ Ph.D. Program for Translational Medicine Taipei Medical University Taipei Taiwan; ^10^ College of Management Taipei Medical University Taipei Taiwan; ^11^ Institute of Clinical Science Zhongshan Hospital Fudan University Shanghai China; ^12^ Department of Radiation Oncology Wanfang Hospital Taipei Medical University Taipei Taiwan; ^13^ Department of Internal Medicine School of Medicine College of Medicine Taipei Medical University Taipei Taiwan; ^14^ Epidemiology and Bioinformatics Center Wanfang Hospital Taipei Medical University Taipei Taiwan

**Keywords:** cancer, case‐control, gender, recurrent aphthous stomatitis, risk factor

## Abstract

**Background:**

Recurrent aphthous stomatitis (RAS) is considered a prophase symptom in patients with specific cancers. This study assessed the association between RAS and subsequent onset of cancer based on a nationwide population‐based database in Taiwan.

**Materials and Methods:**

We selected study participants from the National Health Insurance Research Database from January 2000 to December 2008. Patients in the non‐RAS cohort were matched to case study patients at a 1:1 ratio through frequency matching. All participants were followed up for at least 5 years, and those who received cancer diagnoses during follow‐up were identified.

**Results:**

Among 52 307 patients with and 52 304 patients without RAS, the combined hazard ratio (HR) of all subsequent cancer cases was 1.3 (95% confidence interval [CI]: 1.25‐1.35, *P *=* *0). RAS diagnosis was associated with risk for cancers of the head and neck (aHR = 2, 95% CI: 1.8‐2.3), colon (aHR = 1.2, 95% CI: 1.1‐1.4), liver (aHR = 1.1, 95% CI: 1‐1.3), pancreas (aHR = 1.4, 95% CI: 1.1‐1.7), skin (aHR = 1.4, 95% CI: 1.2‐1.7), breast (aHR = 1.2, 95% CI: 1.1‐1.4), and prostate (aHR = 1.5, 95% CI: 1.3‐1.8), as well as hematologic cancers (aHR = 1.6, 95% CI: 1.3‐1.9). A higher risk was observed for male patients (aHR = 1.35, 95% CI: 1.28‐1.42) than for female patients (aHR = 1.25, 95% CI: 1.18‐1.31) with RAS.

**Conclusions:**

RAS was associated with specific cancers. Susceptible RAS patients should be screened for specific cancers.

## 1 INTRODUCTION

In 2008, 12.7 million cancer cases and 7.6 million cancer deaths were estimated worldwide; however, overwhelming evidence suggests that many malignancies are preventable.^1^, ^2^ Although proportions of patient survival are improving, over half a million people die from cancer each year in the United States alone. Moreover, cancer outranks cardiovascular disease as the number one cause of death in the United States for individuals under the age of 85.^3^ Screening and prevention efforts can reduce mortality due to cancer. Screening detects abnormalities before they are clinically apparent, thereby enabling intervention before cancer has developed or at an early stage, when treatment is most effective.^4^, ^5^ However, susceptible population groups for whom screening should be recommended have not been determined. Easily detectable symptoms or signs among the general population that may predict cancer must be identified so that early cancer screening can be performed.

Recurrent Aphthous Stomatitis (RAS) is one of the most common oral mucosal diseases observed by dental professionals. RAS occasionally coincides with genital mucosa; both are characterized by repeated development of one or multiple discrete painful ulcers that usually heal within 7‐14 days.^6^, ^7^, ^8^, ^9^ Associated lesions are typically circular or oval ulcers 3‐5 mm in length or diameter with a peripheral rim of erythema and a central yellowish adherent exudate. Incidents of RAS range in severity; some patients note only an occasional lesion, whereas others experience continual ulcer activity.^6^, ^7^, ^8^, ^9^ RAS is typically easy to clinically diagnose based on patient history and physical examination.

To date, a possible association between RAS and cancer has never been demonstrated and very few authors have reported as possible.^10^, ^11^, ^12^, ^13^ This is really a new data to report a possible correlation between RAS and cancer risk. To resolve the findings of our clinical practice, we conducted a population‐based case‐control frequency‐matched study to confirm that RAS is a risk factor for specific cancers.

## 2 PATIENTS AND METHODS

### 2.1 Database

Data were collected from the National Health Insurance Research Database (NHIRD) in Taiwan and used to conduct this nationwide population‐based case‐control frequency‐matched cohort study. The NHIRD contains valuable medical records for all individuals under National Health Insurance (NHI) such as registration data, diagnoses, drug prescriptions, expenditure claims in outpatient visits, and inpatient care. The NHIRD is maintained by the NHI program, which is a compulsory program established in 1995 that insures 99% of all residents of Taiwan (approximately 23 million). Hundreds of articles based on NHIRD data have been published in peer‐reviewed international journals^14^, ^15^, ^16^, ^17^, ^18^, ^19^, ^20^, ^21^, ^22^, ^23^, ^24^, ^25^, ^26^ and the detailed data provided in these articles have supported the work of physicians and policymakers.^14^, ^15^, ^16^, ^17^, ^18^, ^19^, ^20^, ^21^, ^22^, ^23^, ^24^, ^25^, ^26^ Individual information was anonymized before the NHIRD was released for research purposes. This study obtained ethical approval from the Institutional Review Board of Taipei Medical University (TMU‐No. 201712019) and was conducted in full compliance with national ethical guidelines.

### 2.2 Selection of study participants

**Figure 1:**
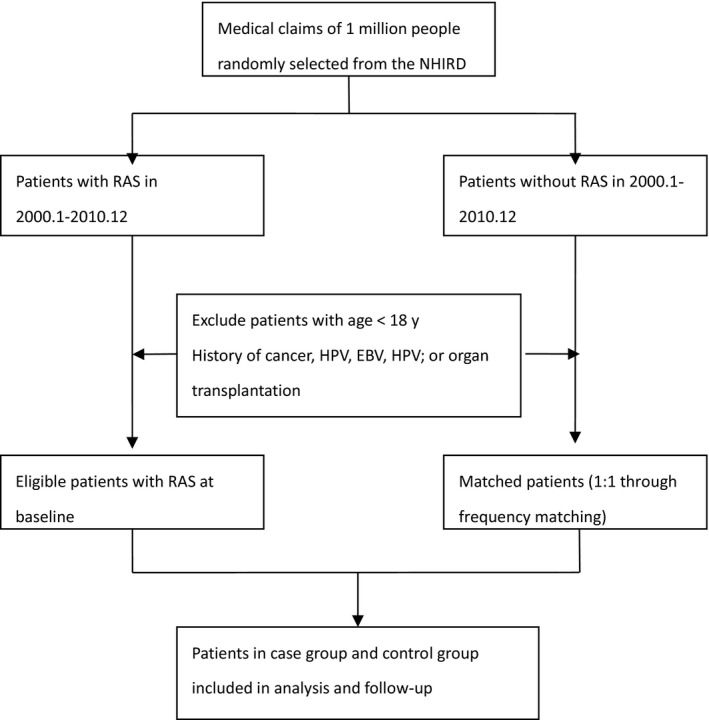
Flow diagram of patient enrollment

Two cohorts were identified for this case‐control study: an RAS cohort and a non‐RAS cohort. A flowchart of the selection procedure is shown in Figure 1. Data regarding patients in the RAS cohort were retrieved from the NHIRD from January 2000 to December 2008. RAS incidence was defined as a minimum of two outpatient visits for oral aphthae (OA; International Classification of Diseases, Ninth Revision, Clinical Modification [ICD‐9‐CM] code 528.2) within 1 year. We excluded patients less than 18 years old and those with human immunodeficiency virus; Epstein‐Barr virus; human papilloma virus; and organ transplantation. To prevent overestimation of the risk of cancer, patients with a history of cancer (ICD‐9‐CM codes 140‐208) were excluded. The index date for each patient in the RAS cohort was designated as their second OA outpatient visit date. Participants in the non‐RAS cohort were selected from patients with no OA outpatient visits or history of cancer. We used frequency matching to match each case patient with one control patient based on year of index date, age group (18‐29, 30‐39, 40‐49, 50‐59, 60‐69, and ≥70 years), sex (male, female), monthly income (NTD0‐15 840, NTD15 841‐25 000, ≥NTD25 001), geographical region (northern, central, eastern, and southern Taiwan), and urbanization level (seven levels, with one being the most urbanized and seven being the least urbanized). For patients in the non‐RAS cohort, index dates were designated as the date of each patient's first use of medical services in the year corresponding with their cohort match's index date year.

### 2.3 Exposure assessment

The primary outcome of interest in this study was cancer diagnosis. Each patient was followed up from index date to the date of receiving a cancer diagnosis or December 2013. Cancer diagnoses recorded for this study comprised 17 common cancers types: head and neck (ICD‐9‐CM codes 140‐149 and 161), esophagus (ICD‐9‐CM code 150), stomach (ICD‐9‐CM code 151), small intestine (ICD‐9‐CM code 152), colon (ICD‐9‐CM codes 153‐154), liver (ICD‐9‐CM code 155), pancreatic (ICD‐9‐CM code 157), lung (ICD‐9‐CM code 162), skin (ICD‐9‐CM code 173), breast (ICD‐9‐CM code 174), uterine (ICD‐9‐CM codes 180‐184), prostate (ICD‐9‐CM code 185), bladder (ICD‐9‐CM code 188), kidney (ICD‐9‐CM code 189), brain (ICD‐9‐CM code 191), thyroid (ICD‐9‐CM code 193), and hematological (ICD‐9‐CM codes 200‐208) cancers.

### 2.4 Statistical analysis

The data analysis in this study was performed using SAS software version 9.2 (SAS Institute, Cary, NC). *P *≤* *0.05 was considered statistically significant. We conducted a descriptive analysis of demographic characteristics, namely age, sex, monthly income, geographical region, and urbanization level. Major comorbidities exhibited within 12 months preceding the index date were also evaluated, namely hypertension (ICD‐9‐CM codes 401‐405), hyperlipidemia (ICD‐9‐CM code 272), and diabetes (ICD‐9‐CM code 250). Chi‐squared tests were conducted to compare the distributions of these characteristics between the RAS and non‐RAS cohorts. The risk of each cancer in the RAS cohort compared with that in the non‐RAS cohort was determined through estimation of the adjusted hazard ratio (aHR) and 95% confidence interval (CI) using a Cox proportional hazard regression model where the hazard ratio (HR) was adjusted for all baseline characteristics and comorbidities. The age‐ and sex‐specific risks were also estimated for both cohorts to enable comparison of subgroup risks.

## 3 RESULTS

This case‐control frequency‐matched study analyzed data of 52 307 patients with RAS and 52 304 patients without RAS. Table 1 lists the demographic characteristics and comorbidities for both cohorts. After application of the frequency‐matching method for the controls, no statistically significant differences were found within the subgroups in terms of age (*P *=* *1), sex (*P *=* *0.996), monthly insured income (*P *=* *1), geographical region (*P *=* *1), or urbanization level (*P *=* *1). The prevalences of comorbidities differed significantly between the two cohorts; patients in the RAS cohort were more likely to have hypertension (24.8% for case vs 23.1% for control, *P *<* *0.001), hyperlipidemia (19.2% for case vs 15.3% for control, *P *<* *0.001), and diabetes (4.4% for case vs 3.9% for control, *P *<* *0.001) than were patients in the non‐RAS cohort.

**Table 1:** Demographic characteristics of patients with recurrent aphthous stomatitis and controls

Factor	No. of cases (%)	No. of controls (%)	*P* value
Overall	52 307	52 304	
Age (y)			
18‐29	9250 (17.7)	9250 (17.7)	>0.99
30‐39	9697 (18.5)	9695 (18.5)	
40‐49	10 674 (20.4)	10 673 (20.4)	
50‐59	9444 (18.1)	9444 (18.1)	
60‐69	6548 (12.5)	6548 (12.5)	
≥70	6694 (12.8)	6694 (12.8)	
Sex			
Male	22 422 (42.9)	22 420 (42.9)	>0.99
Female	29 885 (57.1)	29 884 (57.1)	
Monthly insured income			
≤NT$15 840	23 576 (45.1)	23 576 (45.1)	>0.99
NT$15 841‐25 000	18 155 (34.7)	18 154 (34.7)	
≥NT$25 001	10 576 (20.2)	10 574 (20.2)	
Geographical region			
Northern Taiwan	23 808 (45.5)	23 808 (45.5)	>0.99
Central Taiwan	13 788 (26.4)	13 788 (26.4)	
Southern Taiwan	13 699 (26.2)	13 699 (26.2)	
Eastern Taiwan	1008 (1.9)	1008 (1.9)	
Urbanization level			
1 (most urbanized)	17 657 (33.8)	17 657 (33.8)	>0.99
2	15 060 (28.8)	15 060 (28.8)	
3	8801 (16.8)	8801 (16.8)	
4	6825 (13)	6825 (13)	
5	580 (1.1)	580 (1.1)	
6	1450 (2.8)	1450 (2.8)	
7	1930 (3.7)	1930 (3.7)	
Comorbidity			
Hypertension	12 990 (24.8)	12 072 (23.1)	<0.001
Hyperlipidemia	10 028 (19.2)	8025 (15.3)	<0.001
Diabetes	2286 (4.4)	2016 (3.9)	<0.001

Table 2 shows comparisons of cancer incidence prevalence between the RAS and non‐RAS cohorts. Compared with the non‐RAS cohort, the combined aHR of all cancer outcomes was 1.3 (95% CI: 1.25‐1.35, *P *<* *0.001). RAS was associated with an increased risk for 8 of the 17 cancers studied: head and neck (aHR = 2, 95% CI: 1.8‐2.3, *P *<* *0.001), colon (aHR = 1.2, 95% CI: 1.1‐1.4, *P *<* *0.001), liver (aHR = 1.1, 95% CI: 1‐1.3, *P* = 0.008), pancreatic (aHR = 1.4, 95% CI: 1.1‐1.7, *P* = 0.012), skin (aHR = 1.4, 95% CI: 1.2‐1.7, *P *=* *0.001), breast (aHR = 1.2, 95% CI: 1.1‐1.4, *P *<* *0.001), prostate (aHR = 1.5, 95% CI: 1.3‐1.8, *P *<* *0.001), and hematologic (aHR = 1.6, 95% CI: 1.3‐1.9, *P *<* *0.001) cancers. RAS was not significantly associated with the other nine cancers: esophagus (*P* = 0.255), stomach (*P *=* *0.95), small intestine (*P *=* *0.634), lung (*P *=* *0.79), uterine (*P *=* *0.884), bladder (*P *=* *0.933), kidney (*P *=* *0.562), brain (*P *=* *0.078), and thyroid (*P *=* *0.410) cancers.

**Table 2:** Prevalence of specific cancers among patients with RAS and controls throughout the 5‐y follow‐up

Cancers (ICD‐9‐CM code)	Patients with RAS, n (%)	Controls, n (%)	HR (95% CI)	*P* value	aHR (95% CI)	*P* value
Overall	5987 (11.4)	4861 (9.3)	1.29 (1.241, 1.339)	<0.001	1.3 (1.249, 1.347)	<0.001
Head and neck (140‐149, 161)	801 (1.5)	401 (0.8)	2.1 (1.8, 2.3)	<0.001	2 (1.8, 2.3)	<0.001
Esophagus (150)	104 (0.2)	87 (0.2)	1.2 (0.9, 1.6)	0.16	1.2 (0.9, 1.6)	0.26
Stomach (151)	261 (0.5)	259 (0.5)	1 (0.9, 1.2)	0.66	1 (0.8, 1.2)	0.95
Small intestine (152)	35 (0.1)	31 (0.1)	1.2 (0.7, 1.9)	0.53	1.1 (0.7, 1.8)	0.63
Colon (153 & 154)	1056 (2)	850 (1.6)	1.3 (1.2, 1.4)	<0.001	1.2 (1.1, 1.4)	<0.001
Liver (155)	801 (1.5)	705 (1.3)	1.2 (1.1, 1.3)	0.002	1.1 (1, 1.3)	0.008
Pancreatic (157)	162 (0.3)	119 (0.2)	1.4 (1.1, 1.8)	0.004	1.4 (1.1, 1.7)	0.01
Lung (162)	39 (0.1)	36 (0.1)	1.1 (0.7, 1.7)	0.66	1.1 (0.7, 1.7)	0.79
Skin (172 & 173)	223 (0.4)	157 (0.3)	1.5 (1.2, 1.8)	<0.001	1.4 (1.2, 1.7)	0.001
Breast (174)	674 (1.3)	551 (1.1)	1.3 (1.1, 1.4)	<0.001	1.2 (1.1, 1.4)	<0.001
Uterine (180‐184)	380 (0.7)	383 (0.7)	1 (0.9, 1.2)	0.84	1 (0.9, 1.2)	0.88
Prostate (185)	524 (1)	335 (0.6)	1.6 (1.4, 1.9)	<0.001	1.5 (1.3, 1.8)	<0.001
Bladder (188)	227 (0.4)	223 (0.4)	1.1 (0.9, 1.3)	0.59	1 (0.8, 1.2)	0.93
Kidney (189)	202 (0.4)	188 (0.4)	1.1 (0.9, 1.4)	0.29	1.1 (0.9, 1.3)	0.56
Brain (191)	115 (0.2)	90 (0.2)	1.3 (1, 1.7)	0.05	1.3 (1, 1.7)	0.08
Thyroid (193)	151 (0.3)	138 (0.3)	1.1 (0.9, 1.4)	0.33	1.1 (0.9, 1.4)	0.41
Hematologic (200‐208)	356 (0.7)	226 (0.4)	1.6 (1.4, 1.9)	<0.001	1.6 (1.3, 1.9)	<0.001

aHR, adjusted hazard ratio; CI, confidence interval; HR, hazard ratio; ICD‐9‐CM, International Classification of Diseases, Ninth Revision, Clinical Modification; RAS, recurrent aphthous stomatitis.

All variables in Table 1 were used in the multivariate analysis.

We further analyzed the risk for all cancers based on age and sex stratifications. Regarding age, the highest HR was found for patients aged 50‐59 years (aHR = 1.36, 95% CI: 1.25‐1.47, *P *<* *0.001), followed by those aged 18‐29 years (aHR = 1.35, 95% CI: 1.10‐1.66, *P* = 0.040) and those aged 40‐49 years (aHR = 1.33, 95% CI: 1.21‐1.47, *P *<* *0.001) (Table 3). In men and women, RAS was a significant risk factor for cancer; a higher risk was found for men (aHR = 1.35, 95% CI: 1.28‐1.42, *P *<* *0.001) than for women (aHR = 1.25, 95% CI: 1.18‐1.31, *P *<* *0.001).

**Table 3:** Subgroup analysis of prevalence and HR of cancers among patients with RAS and controls

	Patients with RAS, n (%)	Controls n (%)	Crude HR (95% CI)	*P* value	aHR (95% CI)	*P* value
Age (y)						
18‐29	215 (2.3)	165 (1.8)	1.36 (1.107, 1.661)	0.003	1.35 (1.103, 1.655)	0.004
30‐39	439 (4.5)	356 (3.7)	1.29 (1.121, 1.483)	<0.001	1.28 (1.116, 1.477)	<0.001
40‐49	925 (8.7)	719 (6.7)	1.34 (1.219, 1.482)	<0.001	1.33 (1.208, 1.469)	<0.001
50‐59	1299 (13.8)	1007 (10.7)	1.36 (1.255, 1.48)	<0.001	1.36 (1.248, 1.472)	<0.001
60‐69	1314 (20.1)	1119 (17.1)	1.25 (1.152, 1.351)	<0.001	1.24 (1.149, 1.349)	<0.001
≥70	1795 (26.8)	1495 (22.3)	1.28 (1.194, 1.37)	<0.001	1.28 (1.193, 1.369)	<0.001
Sex						
Male	3021 (13.5)	2385 (10.6)	1.33 (1.264, 1.407)	<0.001	1.35 (1.277, 1.422)	<0.001
Female	2966 (9.9)	2476 (8.3)	1.25 (1.184, 1.317)	<0.001	1.25 (1.184, 1.317)	<0.001

aHR, adjusted hazard ratio; CI, confidence interval; HR, hazard ratio; ICD‐9‐CM, International Classification of Diseases, Ninth Revision, Clinical Modification; RAS, recurrent aphthous stomatitis.

Cancer (ICD‐9‐CM codes 140‐208).

All variables in Table 1 were used in the multivariate analysis.

## 4 DISCUSSION

Recurrent aphthous stomatitis may be associated with inflammatory bowel disease and celiac disease.^27^ The pathogenesis of RAS remains unclear. One hypothesis is that RAS results from a disorder of cell‐mediated immunity, where subsets of proinflammatory cytokines and T cells accumulate.^10^ Hormonal factors, emotional stress, family history, and trauma may predispose a patient to RAS.^8^ Dysregulation in cell‐mediated immunity and accumulation of subsets of proinflammatory cytokines and T cells may be linked with cancers.^10^ In the presence of tumor necrosis factor‐alpha (TNF‐α) and interleukin (IL)‐6, naïve CD4 T cells may develop into Th22 cells that secrete IL‐22 and TNF‐α.^28^, ^29^ Evidence suggests that Th22 cells are involved in cancers.^28^, ^29^, ^30^ The prevalences of proinflammatory cytokines and Th1‐associated chemokine receptors, as detected in protein levels, are similar in patients with RAS and those with cancer.^10^, ^11^, ^12^ The expression of Th2 cytokine IL‐4 within the lesions of patients with oral ulcers suggests complex antigenic stimuli in patients with RAS.^10^, ^31^ In addition to the immune system's effect on the role of RAS and cancer, family history, trauma, hormonal factors, and emotional stress are associated with RAS and the risk of cancer.^32^, ^33^ A plausible hypothesis is that many cancers arise from areas of inflammation.^34^, ^35^ DNA damage in proliferating cells, through their generation of reactive oxygen and nitrogen species induced by phagocytic cells and leukocytes that are produced normally by these cells during the process of inflammation.^36^ These species react to form peroxynitrite, a mutagenic agent.^36^ Hence, repeated tissue damage and regeneration of tissue, in the presence of highly reactive nitrogen and oxygen species released from inflammatory cells, interacts with DNA in proliferating epithelium resulting in permanent genomic alterations such as point mutations, deletions, or rearrangements. Indeed, p53 mutations are seen at frequencies similar to those in cancers in chronic inflammatory diseases such as rheumatoid arthritis and inflammatory bowel disease.^37^ The strongest association of chronic inflammation with cancers is in colon and liver carcinogenesis arising in individuals with inflammatory bowel diseases.^38^ Some specific cancers such as colon, liver, pancreas, breast, prostate, hematology, head and neck and skin cancers where the inflammatory process is a cofactor in carcinogenesis.^34^, ^35^, ^39^, ^40^, ^41^, ^42^, ^43^ About 15% of the global‐specific cancers burden is attributable to inflammation.^35^ These findings were compatible with our outcomes (Table 2).

Recurrent aphthous stomatitis is usually found in patients with Crohn's disease, systemic lupus erythematosus, and Behçet's disease (BD).^27^, ^44^, ^45^ The underlying cause of RAS is unknown. Similar to autoimmune diseases, RAS may represent aberrant immune system activity resulting from exposure to a possibly infectious agent^46^, ^47^, ^48^, ^49^, ^50^ in patients genetically predisposed to develop RAS.^46^, ^51^, ^52^, ^53^, ^54^ Proposed triggering agents include viral and bacterial antigens and other environmental sources.^46^, ^47^, ^48^, ^49^, ^50^ Genetic predisposition to RAS is likely polygenic. Genetic influences associated with RAS include certain human leukocyte antigens (HLAs), including HLA‐B51; however, both HLA and non‐HLA genes may play a role in the disorder.^46^, ^51^, ^52^, ^53^, ^54^ Studies have suggested that altered innate immune function may contribute to RAS.^55^, ^56^, ^57^, ^58^, ^59^ Specifically, deficiencies in mannose‐binding lectin and alterations in the expression of toll‐like receptors have been investigated.^55^, ^56^, ^57^, ^58^ These studies have demonstrated alterations in the numbers of T‐cell subpopulations, as well as evidence of cellular activation.^10^, ^47^, ^60^, ^61^ Autoreactive T cells appear to play a critical role in the pathogenesis of RAS.^62^ A Th1 predominant response has been observed in many studies on RAS, and some studies have demonstrated evidence of a Th2 response.^10^, ^47^, ^60^, ^61^ RAS may involve a mixture of Th1 and Th2 activity and increased activity of Th17 cells.^63^, ^64^, ^65^ In addition to cellular immune activation, evidence of humoral immune activation has been identified in patients with RAS, and autoantibodies against numerous targets have been described.^47^, ^66^, ^67^ Immune complexes may also contribute to RAS.^47^ Inflammation materials such as polymorphonuclear leukocytes (PMNs) are activated in RAS,^68^, ^69^, ^70^, ^71^ and PMNs exhibit increased motility and enhanced adhesion to endothelial cells in vitro.^72^, ^73^ Endothelial dysfunction is typical in patients with RAS and BD.^74^, ^75^, ^76^, ^77^ In such patients, endothelium‐dependent flow‐mediated dilation is reduced and endothelial activation in affected blood vessels mediates vascular inflammation and thrombosis.^75^, ^76^ The critical role of endothelium in cancer formation is not disputed; that cancer causes a hypercoagulable state is accepted with equal certainty.^78^ Furthermore, evidence suggests that these phenomena are linked and that this link may be causative in that changes to vascular biology increase the risks of thrombosis and cancer.^78^ Epigenetic changes may contribute to the pathophysiology of RAS.^79^, ^80^ Alterations in DNA methylation in immune cells in patients with RAS have been described in multiple studies.^79^, ^80^ In summary, RAS may be highly associated with autoimmune diseases. Genetic influences, such as inflammation status; infections; epigenetic changes; and altered innate, cellular, and humoral immune function, may also play common roles in autoimmune disease and cancer formation.^81^, ^82^

Although the exact pathophysiology of cancers in patients with autoimmune diseases has not been fully determined, several mechanisms have been suggested to contribute to the high susceptibility of distinct groups of patients to specific malignancies.^83^, ^84^ These mechanisms include impaired genetic stability, genetic predisposition, immune dysregulation, impaired clearance of oncogenic viruses, and iatrogenic causes.^85^, ^86^ Similarities in the pathologies of autoimmune diseases and cancer have been noted in studies conducted within the preceding 30 years.^85^, ^86^ Inflammatory cytokines and growth factors mediate cell proliferation, and proteinases—especially the collagenase matrix metalloproteinase‐1 (MMP‐1)—contribute to disease progression by remodeling the extracellular matrix and modulating the microenvironment.^87^, ^88^ Autoimmune disorders may activate stromal cells found in these diseases and cancers.^85^, ^86^, ^87^, ^88^ MMP‐1 was originally thought to function not only to degrade interstitial collagens; in addition, novel roles for MMP‐1 have been revealed, involving G protein‐coupled receptors, namely the chemokine receptor CXCR‐4 and protease‐activated receptor‐1 (PAR‐1). Coordination between MMP‐1 and CXCR4 or stromal‐derived factor‐1 signaling influences the behavior of activated fibroblasts in autoimmune diseases and cancers.^85^, ^86^, ^87^, ^88^ In addition, MMP‐1 is a vital part of an autocrine‐paracrine MMP‐1‐PAR‐1 signal transduction axis—a role that amplifies the potential of MMP‐1 to remodel the matrix and modify cell behavior.^87^, ^88^ Finally, new therapeutic agents directed at MMP‐1 and G protein‐coupled receptors might be emerging. These targets underscore fundamental similarities between autoimmune diseases and some cancers.^87^, ^88^

Randomization is often not feasible or permissible in a general population with a rare endpoint such as incidence of cancer. To resolve the problem of selection bias in a general population with a rare endpoint, we designed a case‐control frequency‐matched study. Frequency matching is a promising tool for reducing selection bias in causal inferences based on observational studies and is particularly useful in secondary data analysis of national databases such as the Centers for Medicare and Medicaid Services.^89^ We matched patients with RAS to those without RAS at a ratio of 1:1 through frequency matching. The potential cancer risk‐confounding factors of age,^90^ sex,^91^, ^92^ monthly insured income,^93^, ^94^ geographical region,^95^ and urbanization level^96^ were considered and matched (Table 1). As listed in Table 1, all *P* values were near 1, thereby demonstrating that our matching results were satisfactory and the confounding factors were controlled effectively.^89^ Hypertension,^97^, ^98^ hyperlipidemia,^99^, ^100^ and diabetes^99^ were considered controversial cancer risk factors and were adjusted, as shown in Table 1.

Table 2 illustrates that compared with the non‐RAS cohort, the combined aHR for all cancers was 1.3 (95% CI: 1.25‐1.35). RAS was associated with an increased risk of head and neck, colon, liver, pancreas, skin, breast, prostate, and hematologic cancers. The present study was the first to demonstrate the association between RAS and risk for specific cancers. Head and neck cancers had the highest aHR (aHR = 2, 95% CI: 1.8‐2.3). This study indicated that RAS is an independent risk factor of head and neck cancers. Our findings regarding head and neck cancers were different from those of Li,^13^ who showed that repetitive dental ulcers (odds ratio = 5, 95% CI = 3.17‐8.28) were correlated with high risk for head and neck cancers.^13^ Our data might be completely new information, because repetitive dental ulcers may be subject to differential misclassification as it is nonspecific in comparison with RAS which are diagnosed clinically. The diagnosis of RAS was more clearly identified in our database. Although we did not identify any studies that assessed the incidence of specific cancers in patients with RAS, RAS might been considered a potential early sign of oral cancer.^101^ The different HR is the ratio of the hazard rates corresponding to the conditions described by two levels of an explanatory variable which affected by the number of events per unit time divided by the number at risk. The time interval (throughout the 5‐year follow‐up) and potential effect of RAS‐related inflammation process might be different to cause various specific cancers. There is no clear evidence to prove the necessary time intervals of cancerization process in various cancers.

Table 3 shows that the highest HR among the age‐stratified subgroups was for patients aged 50‐59 years (aHR = 1.36, 95% CI: 1.25‐1.47). However, no statistically significant differences were identified among the age groups. Thus, independent of patient age, RAS is a risk factor for cancer in men and women. Higher risk was observed for male patients with RAS than for female patients with RAS; however, RAS lesions are more common in women. According to our literature review, the present study was the first to demonstrate that male patients with RAS are at a greater risk of cancers than are their female counterparts. Physicians should pay extra attention to men with RAS and carefully analyze oral examination results for early detection of head and neck cancers. The risk ratios of cancers are similar in all patients with RAS, regardless age. Therefore, although cancer incidence is lower among younger patients (<40 years old) than among older patients,^90^ physicians should remain alert to the risk of the previously mentioned cancers in all patients with RAS.

The strengths of this study are its large sample size and the balance between the frequency‐matched RAS and non‐RAS cohorts. The results suggested that RAS is associated with an increased risk of eight cancers. Head and neck cancers had the highest aHR, and male patients with RAS had a higher cancer risk than did their female counterparts. The risk ratios for cancers were similar across all age groups. The present study was the first to reveal that RAS is an independent risk factor for eight cancers. Our results indicate that close surveillance for head and neck, colon, liver, pancreatic, skin, breast, prostate, and hematologic cancers may benefit patients with RAS regardless of age, especially male patients and with regard to the head and neck area. These findings should be considered in the future clinical practice. Patients with RAS exhibit easily detectable symptoms and signs; therefore, RAS may be an observable predictor of cancers.

Based on our study, physicians should be aware of cancer risks in patients with RAS and should conduct detailed surveillance of these patients’ head and neck, colon, liver, pancreas, skin, breast, prostate, and hematologic areas. Close follow‐up might be necessary for early detection of these eight cancer types. The symptoms and signs of RAS are easily detectable; therefore, attention to RAS may aid early detection of early stage cancers in susceptible populations such as male patients with RAS.

This study had some limitations. First, because all patients with RAS enrolled in this study belonged to an Asian population, the corresponding ethnic susceptibility is unclear; hence, our results should be cautiously extrapolated to non‐Asian populations. Second, the relatively low number of patients with small intestinal and lung cancers may have limited the generalizability of our conclusions. Therefore, to obtain crucial information concerning population specificity and disease occurrence, a large‐scale randomized trial that carefully compares selected patients undergoing suitable surveillance is required. Third, the NHI database does not contain information regarding dietary habits, socioeconomic status, or body mass index, all of which may be cancer risk factors. Fourth, diagnoses of all comorbid conditions were dependent on ICD‐9‐CM codes. Nevertheless, the NHI Administration randomly reviews charts and interviews patients to verify the accuracy of diagnoses, and hospitals with outlier charges or practices are liable to an audit and heavy penalties if malpractice or discrepancies are detected. Fifth, some patients never suffered of RAS in their life only because they never performed a specific oral visit. These findings imply that numbers of RAS patients may be underestimated in the present studies. RAS may be a risk factor for specific cancers only could be underestimated, because less ROA patients were identified. The conclusion would not be overturned. Considering the magnitude of the observed effects in this study, these limitations are unlikely to have affected the outcomes.

## 5 CONCLUSIONS

Recurrent aphthous stomatitis was associated with specific cancers. During treatment, patients should be cautioned regarding these associations, especially with respect to head and neck cancers and risks for male patients with RAS. Susceptible patients with RAS should be screened for specific cancers.

## ETHICS APPROVAL AND CONSENT

Our protocols were reviewed and approved by the Institutional Review Board of Taipei Medical University (TMU‐JIRB No. 201712019).

## AVAILABILITY OF DATA AND MATERIAL

The datasets supporting the conclusions of this article are included within the article and its additional files.

## CONFLICTS OF INTEREST

The author(s) declare no potential conflicts of interest. The dataset(s) supporting the conclusions of this article is (are) included within the article.
